# The within-host fitness of HIV-1 increases with age in ART-naïve HIV-1 subtype C infected children

**DOI:** 10.1038/s41598-021-82293-2

**Published:** 2021-02-04

**Authors:** Pradeep Nagaraja, Bindu P. Gopalan, Reena R. D’Souza, Debolina Sarkar, Niharika Rajnala, Narendra M. Dixit, Anita Shet

**Affiliations:** 1 Department of Chemical Engineering, Indian Institute of Science, Bangalore, Karnataka 560012 India; 2 Division of Infectious Diseases, St. John’s Research Institute, St. John’s National Academy of Health Sciences, Bangalore, India; 3 The University of Trans Disciplinary Health Sciences and Technology, Bangalore, India; 4 University of Oxford, Oxford, UK; 5 Centre for Biosystems Science and Engineering, Indian Institute of Science, Bangalore, India; 6 International Vaccine Access Center, Johns Hopkins Bloomberg School of Public Health, 415 N Washington Street, Baltimore, 21321 USA

**Keywords:** Computational models, HIV infections

## Abstract

As the immune system develops with age, children combat infections better. HIV-1, however, targets an activated immune system, potentially rendering children increasingly permissive to HIV-1 infection as they grow. How HIV-1 fitness changes with age in children is unknown. Here, we estimated the within-host basic reproductive ratio, *R*_0_, a marker of viral fitness, in HIV-1 subtype C-infected children in India, aged between 84 days and 17 years. We measured serial viral load and CD4 T cell counts in 171 children who initiated first-line ART. For 25 children, regular and frequent measurements provided adequate data points for analysis using a mathematical model of viral dynamics to estimate *R*_0_. For the rest, we used CD4 counts for approximate estimation of *R*_0_. The viral load decline during therapy was biphasic. The mean lifespans of productively and long-lived infected cells were 1.4 and 27.8 days, respectively. The mean *R*_0_ was 1.5 in children aged < 5 years, increased with age, and approached 6.0 at 18 years, close to 5.8 estimated previously for adults. The tolerogenic immune environment thus compromises HIV-1 fitness in young children. Early treatment initiation, when the *R*_0_ is small, will likely improve viral control, in addition to suppressing the latent reservoir.

## Introduction

The progression of HIV-1 infection in children follows a trajectory that is different from that in adults^1^. In adults, following infection, viremia rises to an acute infection peak within weeks and then declines rapidly to a set-point, where it remains for years^2^. In children, following vertical transmission, viremia rises to a peak in a few months and then decreases gradually, over several years, to a set-point^3^. The viral loads at the peak and at the set-point are significantly higher in children than in adults^1,3^. Survival is poor among perinatally-infected children compared to adults: over 50% of untreated children die within 2 years of birth^4^, while untreated adults survive over 10 years after infection^5^. While these observations seem to indicate that HIV-1 is more aggressive in children than in adults, evidence also exists to the contrary. The immune environment in children is tolerogenic^1,6^ as innate immune cells produce lower levels of proinflammatory cytokines in children than in adults^7^ and high TGFβ levels drive T cell differentiation into regulatory T cells in the foetus^8^. Most children born to HIV-1-infected mothers do not acquire the infection despite continuous exposure over several months^9^, possibly because of the quiescent nature of the target cells^3^. In addition, whereas transmitted strains in adults tend to have higher fitness than the strains prevalent in the donors, such selection for higher fitness is not seen during transmission to children^10,11^. The initial immune reconstitution following the initiation of antiretroviral therapy (ART) is due more to naïve than memory T cells in children, which is the opposite of the observation in adults^12^. Children may also be better at generating broadly neutralizing antibodies than adults^13^. Children who are slow progressors retain high viral loads in contrast to elite controller adults, disease progression in children has no correlation to protective HLA alleles, and infection dynamics in children have been argued to resemble non-pathogenic SIV infection of some non-human primates^1,3,6^. Thus, whether the in vivo fitness of HIV-1 is greater or smaller in children compared to adults remains unknown.

Here, we addressed this question by estimating the within-host basic reproductive ratio, *R*_0_, in children of varying ages. *R*_0_ is defined as the number of new infected cells produced by one infected cell placed in a wholly susceptible cell population^14^. It is thus a composite marker of the intrinsic replicative ability of the virus, the size and permissiveness of the target cell pool, and the strength of the immune response in clearing the infection. The larger the value of *R*_0_, the more fit the virus is in its environment. In a recent study, we estimated *R*_0_ in HIV-1C infected adults by analysing measurements of viral load changes during first-line ART using a mathematical model of viral dynamics^15^. We performed a similar analysis here using measurements of viral load changes in children of different ages who initiated first-line ART with the aim of understanding early viral dynamics and in vivo viral fitness in young children.

## Materials and methods

### Ethics statement

HIV-1-infected children obtaining care at the Infectious Diseases Clinic at St. John’s Medical College, Bangalore, India were eligible to be included in the pediatric HIV cohort study. The study was approved by the Institutional Ethics Review Board at St John’s Medical College Hospital, Bangalore (IERB study reference number 32/2012). Written informed consent was obtained from the parents or legal guardians of all children who were eligible and willing to participate in the study. For children older than 7 years of age, verbal assent was additionally obtained directly from the children and documented using an assent form approved by the IERB. All the experiments were performed in accordance with relevant guidelines and regulations.

### Treatment protocol

First-line ART regimens consisted of two nucleoside reverse transcriptase inhibitors (NRTIs; zidovudine/abacavir/stavudine/tenofovir with lamivudine) and one non-nucleoside reverse transcriptase inhibitor (NNRTI; nevirapine/efavirenz) or one protease inhibitor (PI; lopinavir with ritonavir) in the form of fixed-dose combination pills. At each visit, ART adherence was measured by means of a pill count, taking into account all drugs within the regimen in the form of dispensed pills, any lost or missed pills, pills obtained from other sources and remaining pills.

### Clinical measurements

All children had a baseline pre-ART blood sample. Following ART initiation, samples were obtained at weeks 1, 2, 4, 12, 28, and every 3–6 months thereafter. Laboratory measurements included CD4 T cell count using the FACSCalibur flow cytometer (BD, USA) and HIV-1 viral load using the Abbott m2000rt system (Abbott Molecular Diagnostics, US). Statistical analysis was performed to examine the dependence of these measurements on age and other correlates.

### Fitting of mathematical model of viral dynamics to viral load data

Viral load decay after treatment initiation followed a biphasic decline, with the first phase lasting approximately 2 weeks, followed by a second phase of slower decline, lasting months. The two phases have been attributed to the loss of productively infected cells and long-lived infected cells, respectively^14–18^. To describe this loss, we followed the mathematical model of viral dynamics developed in Shet et al.^15^:

1 $$\frac{dT}{{dt}} = \lambda - d_{T} T - kVT(1 - \varepsilon )$$

2 $$\frac{dM}{{dt}} = \lambda_{M} - d_{M} T - k_{M} VM(1 - \varepsilon_{M} )$$

3 $$\frac{{dT^{*} }}{dt} = kVT(1 - \varepsilon ) - \delta T^{*}$$

4 $$\frac{{dM^{*} }}{dt} = k_{M} VM(1 - \varepsilon_{M} ) - \delta_{M} M^{*}$$

5 $$\frac{dV}{{dt}} = N\delta T^{*} + N\delta_{M} M^{*} - cV$$

In this model, target CD4 T cells, *T*, and long-lived uninfected cells, *M*, such as macrophages, are produced at rates *λ* and *λ*_*M*_, and die with first order rate constants *d*_*T*_ and *d*_*M*_, respectively. They are infected by free virions, *V*, with second order infection rate constants *k* and *k*_*M*_, giving rise to productively infected cells, T^*^, and long-lived infected cells, M^*^, respectively. ART with reverse transcriptase inhibitors blocks the de novo infection of *T* and *M* with efficacies *ε* and *ε*_*M*_, respectively. (Protease inhibitors, not explicitly modelled here, would render a fraction of progeny virions non-infectious and also yield the same biphasic decline.) Infected cells die with first order rate constants *δ* and *δ*_*M*_, respectively, releasing virions at the burst size *N* per cell. Free virions are cleared with a first order rate constant *c*.

When adherence to treatment is high, so that *ε* = *ε*_*M*_ ≈ 1, the above equations yield, upon invoking the standard quasi steady-state approximation between viral and infected cell populations, the following equation, which describes the biphasic decline of viral load following the initiation of ART^15^:

6 $$V(t) = A\exp \left( { - \delta t} \right) + B\exp \left( { - \delta_{M} t} \right)$$

where *V*(*t*) is the viral load at time *t* from the start of therapy, and *A* and *B* represent the contributions to the pre-treatment viral load from the productively infected and long-lived infected cells. We fit Eq. (6) to viral load data from individual children and estimated *A*, *B*, *δ* and *δ*_*M*_, recognizing that $V(0) = A + B$. A similar expression has been used previously to fit viral load changes during therapy in children in the west^17^. We performed the fitting using the nlsLM routine, which employs a Levenberg–Marquardt type algorithm, in R version 3.5.0.

### Estimation of R_0_

Children were considered ‘amenable’ to accurate *R*_0_ analysis if they had 3 viral load measurements over 6 months after starting ART with no viral rebound during this period, had no baseline drug resistance detected, and had baseline CD4 counts > 100 cells/µL. The latter condition is to ensure that severely progressive disease has not occurred and the above model remains applicable. Following previous analyses of adult HIV-1 infection^14,15,19^, assuming a chronic infection steady state, *R*_0_ can be derived to be

7 $$R_{0} = N(kT_{u} + k_{M} M_{u} )/c$$

here $T_{u} = \lambda /d_{T}$ and $M_{u} = \lambda_{M} /d_{M}$ are the populations of target CD4 T cells and long-lived cells in uninfected individuals. *M*_*u*_ is typically small compared to *T*_*u*_^16^, so that $R_{0} \approx NkT_{u} /c = T_{u} /T_{0}$, where $T_{0} = c/Nk$ is the uninfected target cell population in the chronic infection steady state. Thus, *R*_0_ is the ratio of the concentration of the target CD4 T cells had the individual been uninfected to that in the individual in the chronically infected steady state. We recognize that this method of estimating *R*_0_ uses data in the chronic infection state, where the immune system is fully engaged. *R*_0_ is also estimated using the initial growth rate of the virus, where the immune system may not yet be fully triggered^14^. The two approaches have been argued previously to yield similar estimates^14,15^.

Unlike in adults, where CD4 T cell counts remain constant with age, the concentration of CD4 T cells in uninfected children typically decreases with age starting from birth until about 6 or 7 years of age^20,21^. The decrease, however, occurs over a timescale of years. Thus, the CD4 T cell count can be assumed to be approximately constant in each individual for the present analysis, which lasts the duration of treatment (~ weeks). Accordingly, we let the CD4 T cell counts be fixed in each individual during treatment, but let the counts differ across individuals based on their ages. The target cell concentration in uninfected children declined exponentially with age in children from high-income countries (HIC)^20^, but was better described by a linear variation in Indian children^21^. We fit previously reported data^21^ of CD4 T cell counts (mean ± SD) versus age with a straight line and used the best-fit prediction to estimate, *T*_*u*_, the target cell population (mean ± SD) in uninfected individuals of any given age.

For each infected child, the target cell concentration, *T*_0_, was estimated by subtracting the infected cell concentration from the measured CD4 T cell count at treatment initiation. The infected cell population was predominantly productively infected. From Eq. (6), we recognized that the contribution to the viral load from the latter cells is *A*. Substituting this in Eq. (5) and using the pre-treatment pseudo steady state yielded the productively infected cell concentration as *Ac/Nδ*, where *A* and *δ* were obtained from fits to the viral load data (see above) and *c* = 23/day^22^ and *N* = 50,000 virions/cell^23^ were the viral clearance rate and burst size, respectively. If left untreated, the viral load in children varies but does so slowly, with a timescale of years, until a set-point is reached. Thus, again, the infection may be assumed to be in a pseudo-steady state for a child at any age. *R*_0_ was thus estimated as the ratio of *T*_*u*_ and *T*_0_ for each child amenable to analysis using the model described above. For the rest, approximate *R*_0_ values were obtained as the ratio of *T*_*u*_ and the CD4 T cell count at treatment initiation, recognizing that the latter count was almost entirely of uninfected target cells.

## Results

### Subject characteristics

**Figure 1:**
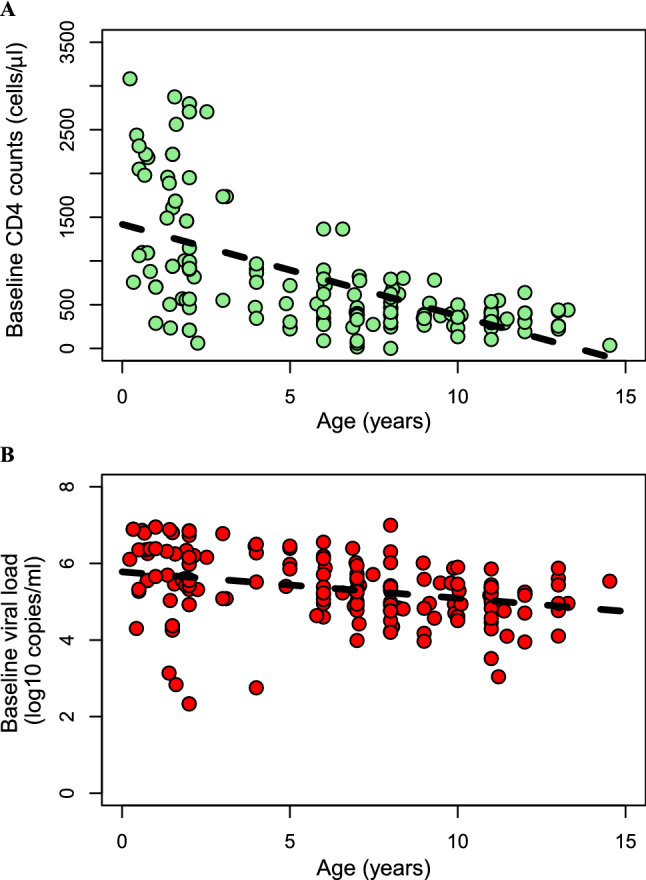
Dependence of baseline characteristics on age. (**A**) Baseline CD4 T cell count decreases significantly with age (Pearson *ρ* = − 0.63; *P* = 2.2 × 10^–16^). (**B**) Baseline viral load also decreases with age (Pearson *ρ* = − 0.313; *P* = 3 × 10^–5^). Each dot represents an HIV-1 infected child (n = 171) and the dashed lines are linear regression lines.

Between 2012 and 2014, 171 HIV-1 infected children aged between 84 days and 17 years, with median age 7.0 years (IQR 2.0–9.9), were enrolled and initiated on first-line antiretroviral therapy. Median baseline viral load and CD4 T cell count were 2.2 × 10^5^ copies/mL (IQR 0.7–9.6 × 10^5^) and 467 cells/μL (IQR 321–913), respectively. The baseline CD4 T cell count and viral load declined significantly with age (Fig. 1), similar to previous observations^24,25^. Most of the children (113 of 117 subtyped) were infected with HIV-1 subtype C. All children had average pill count-based adherence levels greater than 95%.

### Viral decay analysis

Measurements of viral load changes during therapy from 25 children were amenable to analysis using our mathematical model of viral dynamics. Those with fewer than three viral load measurements in 6 months from the onset of therapy (n = 120) or those with viral load rebound within the three viral load measurements (n = 16) were excluded. From the remaining children, those harbouring baseline drug resistance mutations (n = 7), and those with baseline CD4 T cell counts lower than 100 cells/μL (n = 3) were also excluded. The 25 children included in the analysis were all infected with HIV-1C.

**Figure 2:**
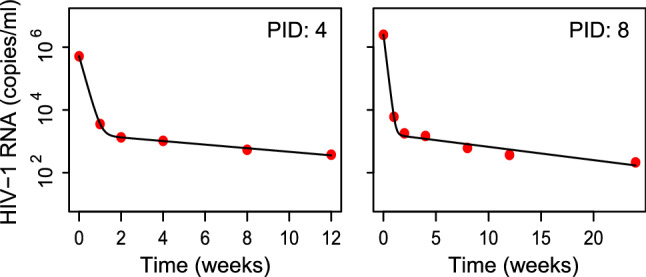
Model fits to data. Representative fits (lines) of model predictions (Eq. 1) to viral load measurements(symbols) in two HIV-1 infected children following the onset of cART. Fits to data from other children are shown in Fig. S1. The best-fit parameter estimates are in Table 1.

Our model (Eq. 6) provided good fits to the biphasic viral load decline observed in each child (Fig. 2, Fig. S1, Table 1). The model parameters, including the best-fits are summarized in Table S1. From the fits, we estimated the first phase slope, *δ*, to be 0.74 ± 0.22/day and the second phase slope, *δ*_*M*_, to be 0.037 ± 0.029/day. (When we restricted the fits to children with at least five viral load measurements (n = 10), the corresponding estimates were 0.7 ± 0.1/day and 0.024 ± 0.012/day, respectively. These variations did not influence our estimates of *R*_0_.) The corresponding lifespans of productively infected cells, *τ*, and long-lived infected cells, *τ*_*M*_, were 1.4 days and 26.8 days, respectively. Neither *δ* nor *δ*_*M*_ was significantly correlated with age (Pearson *ρ* = -0.04 (*P* = 0.85) and *ρ* = 0.024 (*P* = 0.9) for *δ* nor *δ*_*M*_, respectively; Fig. S2). Indeed, the mean *δ*_*M*_ was similar to the 0.03/day estimated recently for HIV-1C infected adults in India^15^ (*P* = 0.2). Further, we found that > 98% of the viral load at baseline was due to virions that originated from productively infected cells, while the remaining 2% of virions came from the long-lived infected cells (Table 1). The baseline viral load did not increase significantly with *δ* (Pearson *ρ* = 0.17 (*P* = 0.43); Fig. S3A) or *δ*_*M*_ (Pearson *ρ* = 0.15 (*P* = 0.48); Fig. S3B).

**Table 1:** Baseline characteristics, best-fit parameter values and estimates of *R*_0_ in Indian children.

Patient ID	Age (years)	Baseline CD4 count (cells/μL)	Baseline viremia (copies/mL)	*A* (copies/mL)	*B* (copies/mL)	*A*/(*A* + *B*) (%)	δ (/day)	δ_M_ (/day)	Baseline infected CD4 count (cells/μL)	CD4 count in age-matched healthy children (cells/μL)	*R*_0_
7^a^	2.5	2706	1,453,953	1,445,009	8944	99.38	0.77	0.095	0.86	1629 ± 577	0.64 ± 0.2
24^a^	3.1	1737	121,363	119,018	2345	98.07	0.72	0.029	0.08	1594 ± 566	0.9 ± 0.3
21	4.9	514	252,728	250,812	1916	99.24	0.75	0.04	0.15	1490 ± 532	2.9 ± 1.0
18	6.6	1366	170,728	163,080	7648	95.52	0.75	0.033	0.1	1392 ± 501	1.0 ± 0.4
8	6.9	244	2,495,636	2,493,867	1769	99.93	0.9	0.014	1.27	1375 ± 495	5.7 ± 2.0
35	6.9	615	76,830	76,285	545	99.29	0.35	0.022	0.1	1375 ± 495	2.2 ± 0.8
10	7	415	1,049,765	1,044,767	4998	99.52	0.55	0.016	0.87	1369 ± 494	3.3 ± 1.2
32	7.1	777	266,009	265,176	833	99.69	0.95	0.061	0.13	1363 ± 492	1.8 ± 0.6
4	7.5	276	516,423	514,698	1725	99.67	0.78	0.019	0.3	1340 ± 484	4.9 ± 1.8
33	8.1	679	22,498	22,205	293	98.7	0.58	0.007	0.02	1306 ± 473	1.9 ± 0.7
23	8.2	623	69,213	68,661	552	99.2	0.59	0.013	0.05	1300 ± 471	2.1 ± 0.8
27	8.4	804	65,000	63,744	1256	98.07	0.87	0.047	0.03	1288 ± 468	1.6 ± 0.6
3	9	347	1,030,471	1,021,087	9384	99.09	0.69	0.024	0.68	1254 ± 457	3.6 ± 1.3
34	9	391	386,682	383,987	2695	99.3	0.76	0.028	0.23	1254 ± 457	3.2 ± 1.2
30	9.2	518	90,862	90,445	417	99.54	1.56	0.001	0.03	1242 ± 453	2.4 ± 0.9
31	9.3	782	37,454	36,544	910	97.57	0.51	0.042	0.03	1236 ± 451	1.6 ± 0.6
13	9.5	375	310,292	306,801	3491	98.87	0.88	0.112	0.16	1225 ± 447	3.3 ± 1.2
19	9.8	398	309,266	307,456	1810	99.41	0.94	0.026	0.15	1208 ± 442	3.0 ± 1.1
2	9.9	372	87,137	86,720	417	99.52	0.77	0.012	0.05	1202 ± 440	3.2 ± 1.2
11	9.9	406	50,742	50,414	328	99.35	0.61	0.014	0.04	1202 ± 440	3.0 ± 1.1
16	9.9	262	754,240	751,684	2556	99.66	0.7	0.014	0.49	1202 ± 440	4.6 ± 1.7
9	10	401	193,059	185,310	7749	95.99	0.65	0.051	0.13	1196 ± 438	3.0 ± 1.1
25	10.1	382	86,661	85,084	1577	98.18	0.64	0.05	0.06	1190 ± 436	3.1 ± 1.1
15	11	300	147,678	144,969	2709	98.17	0.58	0.078	0.11	1138 ± 420	3.8 ± 1.4
36	12	193	176,653	174,978	1675	99.05	0.57	0.086	0.14	1081 ± 401	5.6 ± 2.1
Mean						98.80	0.74	0.037			
SD						1.10	0.22	0.029			

Age, baseline CD4 counts and viremia of the 25 children analyzed using Eq. (1) are listed. The best-fits (Fig. S1) yielded values of *A* and *B*, which mark the contributions to the baseline viremia from productively infected and long-lived infected cells, respectively. 100 × *A/A* + *B* thus yields the percentage of the virions at baseline that originated from productively infected cells. The fits also yielded the loss rates of productively infected cells, *δ*, and long-lived infected cells, *δ*_*M*_. Uncertainties in estimates of *A*, *δ*, and *δ*_*M*_ are in Table S4. Subtracting the infected cell count at baseline (*Ac/Nδ*) from the CD4 count yielded an estimate of the uninfected target CD4 count at baseline (see “Methods”). Dividing the CD4 count in healthy individuals of the same age (Fig. S4) with the latter uninfected target CD4 count yielded the basic reproductive ratio, *R*_0_. Means and standard deviations are reported for quantities that are not correlated with age.

^a^The baseline CD4 count in these very young children was larger than the mean count in healthy children, resulting in estimates of *R*_0_ < 1.

### Basic reproductive ratio, ***R***_0_

**Figure 3:**
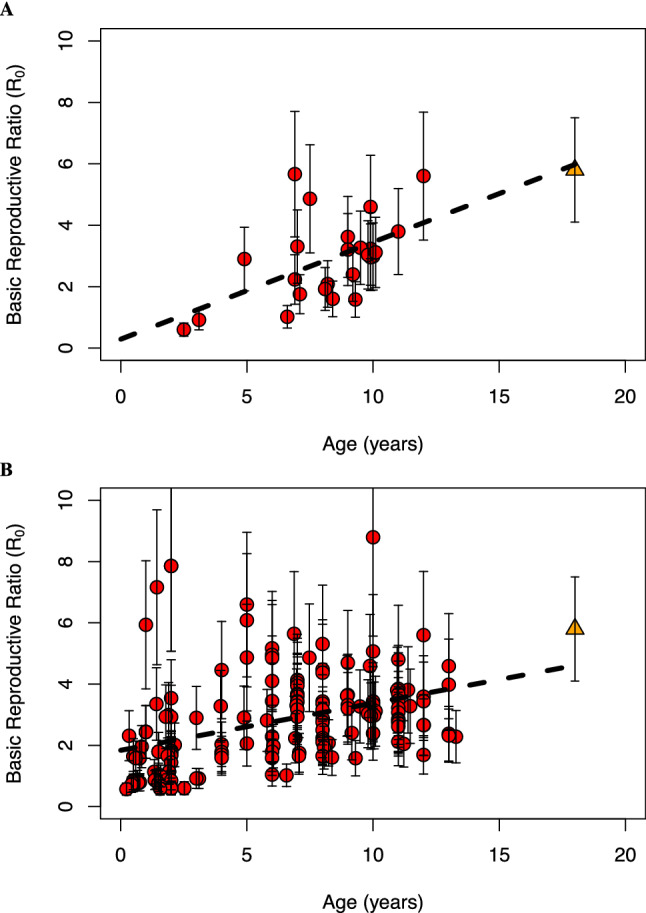
Dependence of *R*_0_ on age. (**A**) Estimates of *R*_0_ for the children analyzed using the mathematical model of viral dynamics (n = 25, listed in Table 1). (**B**) Approximate estimates of *R*_0_ from all children with baseline CD4 T cell counts above 100 cells/µL (n = 157, see text). Red circles indicate the mean *R*_0_ for an individual. Error bars represent *R*_0_ estimated using CD4 T cell counts 1 SD above and below the mean, measured in healthy Indian individuals^21^. The orange triangle is the mean *R*_0_ for HIV-1C in Indian adults estimated previously^15^. Error bar represents the standard deviation. Dashed line is the best-fit line. Pearson ρ = 0.53 (*P* = 0.006) in (**A**) and Pearson ρ = 0.38 (*P* = 9.1 × 10^–7^) in (**B**).

The CD4 T cell counts displayed a linear age-dependent decline in uninfected Indian children^21^. A linear fit yielded $CD4\left( a \right) = 1773 - 58a$, where *CD*4(*a*) is the mean CD4 T cell count (in cells/µL) at age *a* (in years) (Fig. S4). We repeated the fits also for values one SD removed from the mean (Fig. S4). From the viral load data analysis using our mathematical model of viral dynamics above, we estimated the uninfected target CD4 T cell count in each infected child at baseline. The ratio of CD4 (*a*) and the latter count yielded *R*_0_ (Table 1). We found that *R*_0_ increased significantly with age (Pearson ρ = 0.53 (*P* = 0.006); Fig. 3A). For age < 5 years, the estimated mean *R*_0_ was 1.5 (n = 3), and this value increased to 3.1 in children aged 5–8 years (n = 6), and further to 4.2 in children aged 10–12 years (n = 3). Remarkably, when the linear regression of *R*_0_ with age was extrapolated to the age of adulthood, i.e., 18 years, the predicted *R*_0_ was 6.0, which was close to the value of 5.8 ± 1.7 estimated previously in adults^15^ (Fig. 3A), giving us confidence in our analysis.

For children not amenable to analysis using the mathematical model of viral dynamics, due, for instance, to too few viral load measurements or viral rebound (see above), we obtained approximate estimates of *R*_0_. The infected CD4 T cells typically form a negligible fraction (< 0.1%) of the total CD4 T cell count (compare *Ac/Nδ* with the baseline CD4 count in Table 1; also see Ref.^15^), allowing us to use the baseline CD4 T cell measurements as a good approximation of the target cell pool. Dividing the expected CD4 T cell count in a healthy individual of the same age with the baseline CD4 T cell count thus yielded approximate values of *R*_0_. Excluding children infected with non-C subtypes and those with baseline CD4 counts below 100 cells/µL, we obtained these estimates for 157 children (Table S2 and Fig. 3B). *R*_0_ values estimated by this ‘approximate’ method (Fig. 3B) were similar to those obtained using our mathematical model of viral dynamics (Table S3), and were again correlated with age (Pearson ρ = 0.38 (*P* = 9 × 10^–7^)). (Excluding the 22 children with known resistance mutations at baseline did not alter our conclusions; Pearson ρ = 0.36 (*P* = 2 × 10^–5^).) The increase with age was also evident when *R*_0_ was stratified in one-year bands (Fig. S5). (Note that in the very young children (< 2 year-old), a few children had *R*_0_ < 1. We expect this to be due to the uncertainty in the CD4 T cell counts in the healthy individuals of the same age group. Because measurements of CD4 T cell counts were not available, we have used a linear extrapolation from measurements in higher age groups (Fig. S4). A higher CD4 T cell count than estimated by such an extrapolation, which may be expected based on measurements in children in the west^20^, would yield *R*_0_ > 1 in these individuals.) Extrapolating a linear fit to 18 years of age yielded estimates close to that in adults, reinforcing our findings above.

We recognized that HIV-1 infection can compromise thymic output^12,26^, which would manifest, among other things, as a lower target cell pool in infected individuals than what is expected from age-matched uninfected individuals. Despite important advances, combining experiments and modelling, to account for the effects of HIV-1 and antiretroviral treatment on thymic output, uncertainties remain^27–32^. Peripheral proliferation, for instance, has been argued to influence CD4 T cell counts together with the reduction in thymic output, with the latter not strongly dependent on age^31,32^. CD4 T cell counts are not restored to levels in uninfected children even after therapy establishes viremic control, the cause of which remains to be elucidated^30^. Accounting accurately for the effects of HIV-1 infection on thymic output awaits future studies that would resolve the above uncertainties. Here, we examined whether these effects would alter our findings qualitatively. Current estimates suggest a reduction in thymic output in HIV-1-infected individuals to ~ 30% of that in uninfected individuals^28^. This would amount to a corresponding reduction of the target cell pool and hence *R*_0_. Using the latter as conservative estimates, we reestimated *R*_0_ and found that although the absolute values were expectedly lower, the trends of *R*_0_ increasing with age remained (Fig. S6).

Our estimated *R*_0_ values (Fig. 3A,B) thus showed a consistent increase with age that suggested that the changing host environment confers increasing fitness on HIV-1 as children grow from infancy to young childhood and subsequently to adolescence.

## Discussion

The interaction between HIV-1 and the host is complex in children because of the ongoing development of the immune system. The immune system undergoes a transition from being tolerogenic at birth to being more immunogenic in adolescence^6^. A tolerogenic environment may be conducive to host survival despite high viremia, as has been observed in the non-pathogenic SIV infection of natural non-human primate hosts^33^. Akin to these non-human primate hosts and unlike adult humans, HIV-1 infected children have high viremia^1^. Yet, the gut in children, which is replete with highly susceptible Th17 cells, is more prone to microbial translocation compared to the gut in adults or natural primate hosts, leading to a greater degree of immune activation and disease progression^1^. Mortality in HIV-1 infected children is higher than adults^4,5^. Whether HIV-1 is more fit in children than in adults thus remained unclear. Here, we argue that the host environment renders HIV-1 with a greater fitness with increasing age in children. Using principles similar to a previous analysis in adults^15^, we estimated the within-host basic reproductive ratio, *R*_0_, of HIV-1 in children and found that it increased with age in children and eventually approached the value in adults.

*R*_0_ is a metric of viral fitness that integrates the influence of viral and host factors in determining the ability of the virus to spread within an infected individual. We therefore employ it as a single, combined metric to assess within-host viral fitness. (Alternative metrics of fitness based on other aspects of the infection, such as intrinsic replicative ability or between-host transmission, have been proposed^34^.) The combination of viral and host factors implies, for instance, that a strain with a higher intracellular replicative ability may have a lower *R*_0_ than a strain with a lower replicative ability if target cells are more susceptible to the latter. Indeed, we found previously that adult HIV-1C infection in India had a lower *R*_0_ than HIV-1B in the west despite set point viremia being higher with the former^15^. We argued that this difference arose from the different infectivity and replicative ability of the two strains. A previous longitudinal study has argued that the intrinsic replicative ability of HIV-1 does not change with age in children^36^. The increase in *R*_0_ we estimate must thus arise from a change in host factors with age. Immune activation levels rise with age in children^6^. This may render target cells more susceptible to infection. The increased susceptibility appears to offset the decrease in the CD4 T cell concentration with age. It is important to note here that although the concentration of CD4 T cells decreases, the total CD4 T cell population in the body rises significantly with age because the blood volume undergoes a 15–20 fold increase from birth to adolescence^31^. Whether this increases local densities of target cells in lymphoid tissues, where the infection is thought to spread, remains to be examined. Nonetheless, the greater absolute target cell population may contribute in a subtle way, via system size effects, to the rise of *R*_0_ with age. Accounting for system size effects, which becomes important when the sizes are small, is beyond the scope of our study. Other changes in the host that reduce the tolerogenic nature of the immune system, such as the production of higher levels of proinflammatory cytokines^7^, may also contribute to the increase in *R*_0_ with age.

Our analysis of viral load changes during ART yielded estimates of the half-lives of productively infected cells. The half-lives did not depend significantly on age, consistent with previous reports^18^. The loss of infected cells occurs due to viral cytopathicity as well as immune mediated killing, the latter exhibiting complex characteristics in adults^35^. That infected cell half-lives did not decrease significantly with age, when the immune system is expected to increase its aggressiveness, suggests that cell death was primarily due to viral cytopathicity in the children we studied. That the viral replicative capacity is thought not to change with age^36^ is thus consistent with viral cytopathicity and hence infected cell half-life being independent of age. Indeed, the half-lives of long lived infected cells we estimated were not different from those in adults^15^. The increased immune activation with age thus appears to contribute primarily to increasing the activation level and hence susceptibility of target cells.

The half-lives of productively infected cells in Indian children, infected with HIV-1C, were comparable to those in western children, infected with HIV-1B. The mean half-life we estimated here was 1.4 days. In western children, a range of estimates is reported based on the treatment employed. Early estimates, possibly with less efficacious therapy and less frequent sampling than in more recent studies, were 2.3 days^17^. The estimates become shorter with more potent treatment, approaching 1.2 days in 31 children with high dose ritonavir-based highly active ART (HAART)^18^ and 0.9 days in six children with mega-HAART^37^. We recognize that our sampling frequency, where the first data point after treatment initiation is at week 1, could lead to an underestimation of the first phase slope, in which case, the half-life of productively infected cells would be shorter than 1.4 days. The half-lives of long-lived infected cells are more difficult to compare because of the wide range reported in studies on western children, although our estimate lies within this range. Whereas our present estimate is a mean of 27 days, the estimates in western children range from 9 to 43 days^17,18,37^.

We estimated *R*_0_ as the ratio of the CD4 T cell counts in uninfected children and the uninfected target CD4 T cell counts in infected children of the same age. The infected CD4 T cell numbers form a minuscule fraction of the total CD4 T cell count in infected individuals (see Table 1) implying that the measured CD4 T cell counts are a reasonably accurate approximation to the uninfected target CD4 T cell counts in the latter individuals. CD4 T cell counts in healthy children decrease with age. Thus, for *R*_0_ to increase with age, the CD4 T cell counts in infected individuals must decline with age faster than in uninfected individuals. The decline may, at least in part, be due to the reduction in thymic output due to the infection^28^. Regardless, with each passing year, thus, the loss of CD4 T cells due to the infection gets larger and increasingly consequential, leading ultimately to disease progression.

When ART with an efficacy ε is used, *R*_0_ drops during therapy to *R*_t_ = *R*_0_(1 − ε)^14^. The goal of therapy is to drive *R*_t_ to below 1. The minimum ε that ensures this *R*_t_ value is termed the critical efficacy, ε_c_, and equals 1 − 1/*R*_0_^15,38^. Thus, the larger the *R*_0_, the greater is ε_c_ and hence more stringent is the demand on ART in terms of combination drugs, adherence and dosage. Because *R*_0_ increases with age, treating children earlier would achieve the desired virologic outcome more efficiently. Early initiation of ART thus provides the advantage of more rapidly lowering viral load in addition to reducing the size of the latent reservoir^39^. Other considerations, including the difficulties in dosing very young children, could confound these inferences.

Given that a significant fraction of the children infected at birth and untreated die within the first 2 years^4^, the older children in our analysis are likely to be survivors and, to some extent, slow progressors. Whether this results in a bias towards lower values of *R*_0_ remains to be ascertained. *R*_0_ is likely to be higher in children who initiated treatment under 2 years of age but were otherwise expected to progress rapidly. The fates of children who initiated treatment under 2 years of age in our study were not known. Nonetheless, our finding that the estimated pediatric *R*_0_ values transitioned smoothly with age to our previously estimated adult values^15^ gave us confidence in our estimates.

We recognize potential approximations introduced by our method of estimating *R*_0_. Using the ratio of target CD4 T cell populations in uninfected and chronically infected individuals would yield *R*_0_ contingent on the establishment of the chronic infection steady state. Although the viral loads remain steady in chronically infected adults, the CD4 T cell counts gradually decline as the disease progresses, the predominant reason for which is argued to be pyroptosis^40^. When an abortive infection of a cell occurs, due, for instance, to imperfect reverse transcription, the cell experiences pyroptosis driven by the caspase-1 pathway. This process causes the secretion of pro-inflammatory cytokines such as IL-1β, which create a heightened inflammatory state, recruiting more CD4 T cells to the sites of inflammation, increasing infection and cell death^41^. This could introduce a bias in our estimates of *R*_0_ because in our model, a decrease in CD4 T cell counts would manifest as an increase in *R*_0_. We expect this bias, however, to be small for two reasons. First, the immune activation levels in HIV-1 infected children have been found to be significantly lower than in adults^42^, suggesting that pyroptosis may have a much weaker role in children than adults. In our study, because we considered children older than 2 years, there would be an enriching of slow progressors, arguing further for low immune activation levels, akin to non-pathogenic infection of monkeys^43^. Second, a more accurate method for estimating *R*_0_ is based on analysing viral growth rates during acute infection^44^, which we found yielded estimates for adult HIV-1 subtype B infection not different from that obtained using our procedure^15^. We understand this equivalence as follows: when chronic infection is established, CD4 T cell counts drop quickly below the uninfected state, to a level determined largely by *R*_0_. They drop further slowly, driven by pyroptosis. The duration of infection at the time CD4 T cell counts are measured in the chronic steady state is typically not known in untreated individuals. The variation in CD4 T cell counts across individuals can be large (1039 ± 315 cells/μL in healthy South Indian adults^45^), which clouds the variation introduced by pyroptosis. Indeed, randomly sampling CD4 T cell numbers from the latter distribution of CD4 T cell counts in South Indian adults as an estimate of the CD4 T cell counts in the uninfected state for each individual, yielded average estimates of *R*_0_ no different from that obtained using the mean CD4 T cell counts. We therefore expect our method of estimating *R*_0_ to be reliable.

Models that include the variation of thymic output with age and the influence HIV infection has on thymic lymphocyte numbers have been constructed^46^. Applying those models to the analysis of short-term viral load changes during therapy is difficult given the large number of unknown parameters involved. Incorporating the approximate effects of the variations in thymic output, which made analysis possible with our model, did not alter our findings qualitatively. The more involved transition from the tolerogenic to the immunogenic nature of the immune system is likely to influence *R*_0_ significantly; a robust description of the transition that can be incorporated into models, possibly building on recent techniques to quantify tolerance^47^, awaits future studies.

In summary, our study argues that as HIV-1 progresses in children, there is increased in vivo viral fitness with age. Early treatment initiation may therefore elicit enhanced and more sustained responses. The increase in fitness with age appears to arise less from viral evolution and more from the developmental processes, particularly those associated with the immune system, in children. Future studies may quantify these developmental processes and their contribution to HIV-1 fitness.

## Supplementary Information

Supplementary Information.

## Data Availability

The data and the models in the manuscript are available upon request to the corresponding authors by email.
