# Supplementary material for: The within-host fitness of HIV-1 increases with age in ART-naïve HIV-1 subtype C infected children
Source: Sci Rep. 2021 Feb 4;11:2990. doi: 10.1038/s41598-021-82293-2 (PMC7862260; doi:10.1038/s41598-021-82293-2)
Supplement: Supplementary file 1 — Supplementary Information. [file 41598_2021_82293_MOESM1_ESM.pdf]

Supplementary Information

The within-host fitness of HIV-1 increases with age in ART-naïve HIV-1 subtype C infected children

Pradeep Nagaraja[1], Bindu P Gopalan[2,3] Reena R D'Souza[2,4], Debolina Sarkar[1], Niharika Rajnala[2], Narendra M Dixit[1,5], Anita Shet[6]

[1]Department of Chemical Engineering, Indian Institute of Science, Bangalore

[2]Division of Infectious Diseases, St John's Research Institute, St John's National Accademy of Health Sciences, Bangalore, India

[3]The University of Trans Disciplinary Health Sciences and Technology, Bangalore, India

[4]University of Oxford, United Kingdom

[5]Centre for Biosystems Science and Engineering, Indian Institute of Science, Bangalore

[6]International Vaccine Access Center, Johns Hopkins Bloomberg School of Public Health, Baltimore, USA

Table S1. Parameter values used in the study.

Symbol	Meaning	Value or range	Source
N	Burst size	50000 virions/cell	Ref. 22
c	Viral clearance rate	23/day	Ref. 21
δ	Death rate of productively infected cells	0.35-1.56/day	Best-fit (Table 1)
δ_M	Death rate of long-lived infected cells	0.001-0.112/day	
A	Viral load contribution from productively infected cells at baseline	2.2×10^4-2.5×10^6 copies/ml	
B	Viral load contribution from long-lived infected cells at baseline	293-9384 copies/ml	

Table S2. Approximate estimates of R_0 in Indian children. Age, baseline CD4 counts in infected children (n=157) and baseline CD4 counts in age-matched healthy children (Fig. S4) are reported. Dividing the CD4 count in healthy individuals of the same age with the baseline CD4 count in infected children yielded the basic reproductive ratio, R_0.

**Age (y)**	**Baseline CD4 count (cells/µl)**	**Estimated CD4 counts in age-matched healthy individuals (cells/µl)**	**Approximate estimate of R_0**
0.2	3083	1760±619	0.6±0.2
0.3	758	1754±617	2.3±0.8
0.4	2439	1748±615	0.7±0.3
0.5	1063	1744±614	1.6±0.6
0.5	2050	1744±614	0.9±0.3
0.5	2315	1744±614	0.8±0.3
0.6	1098	1739±612	1.6±0.6
0.7	1981	1735±611	0.9±0.3
0.7	2219	1733±610	0.8±0.3
0.8	1094	1730±609	1.6±0.6
0.8	1094	1730±609	1.6±0.6
0.8	2183	1729±609	0.8±0.3
0.8	881	1725±608	2±0.7
1	289	1715±605	5.9±2.1
1	702	1715±605	2.4±0.9
1	704	1715±605	2.4±0.9
1	704	1715±605	2.4±0.9
1.3	1492	1696±598	1.1±0.4
1.4	1958	1695±598	0.9±0.3
1.4	1890	1692±597	0.9±0.3
1.4	505	1691±597	3.3±1.2
1.4	236	1690±596	7.2±2.5
1.5	941	1686±595	1.8±0.6
1.5	941	1686±595	1.8±0.6
1.5	2220	1686±595	0.8±0.3
1.5	2220	1686±595	0.8±0.3
1.5	1609	1686±595	1±0.4
1.6	2876	1683±594	0.6±0.2
1.6	1685	1682±594	1±0.4
1.6	1685	1682±594	1±0.4
1.6	2563	1680±593	0.7±0.2
1.8	569	1670±590	2.9±1
1.9	1005	1666±589	1.7±0.6
1.9	556	1663±588	3±1.1
1.9	1458	1662±588	1.1±0.4
1.9	1458	1662±588	1.1±0.4
2	211	1658±586	7.9±2.8
2	211	1658±586	7.9±2.8
2	468	1658±586	3.5±1.3
2	566	1658±586	2.9±1
2	906	1658±586	1.8±0.6
2	916	1658±586	1.8±0.6
2	916	1658±586	1.8±0.6

2	942	1658±586	1.8±0.6
2	987	1658±586	1.7±0.6
2	993	1658±586	1.7±0.6
2	1150	1658±586	1.4±0.5
2	1953	1658±586	0.8±0.3
2	2706	1658±586	0.6±0.2
2	2706	1658±586	0.6±0.2
2	2797	1658±586	0.6±0.2
2	2797	1658±586	0.6±0.2
2.1	819	1649±583	2±0.7
2.5	2706	1628±576	0.6±0.2
3	552	1600±568	2.9±1
3	1737	1600±568	0.9±0.3
3.1	1737	1594±566	0.9±0.3
4	471	1544±550	3.3±1.2
4	346	1542±549	4.5±1.6
4	758	1542±549	2±0.7
4	865	1542±549	1.8±0.6
4	910	1542±549	1.7±0.6
4	968	1542±549	1.6±0.6
4.9	514	1491±533	2.9±1
5	225	1485±531	6.6±2.4
5	244	1485±531	6.1±2.2
5	305	1485±531	4.9±1.7
5	721	1485±531	2.1±0.7
5.8	511	1438±516	2.8±1
6	276	1427±512	5.2±1.9
6	288	1427±512	5±1.8
6	294	1427±512	4.9±1.7
6	348	1427±512	4.1±1.5
6	414	1427±512	3.4±1.2
6	624	1427±512	2.3±0.8
6	796	1427±512	1.8±0.6
6	898	1427±512	1.6±0.6
6	1366	1427±512	1±0.4
6.1	721	1424±511	2±0.7
6.6	1366	1394±501	1±0.4
6.9	244	1377±496	5.6±2
6.9	615	1374±495	2.2±0.8
7	415	1371±494	3.3±1.2
7	331	1369±494	4.1±1.5
7	339	1369±494	4±1.5
7	347	1369±494	3.9±1.4
7	372	1369±494	3.7±1.3
7	375	1369±494	3.7±1.3
7	381	1369±494	3.6±1.3
7	398	1369±494	3.4±1.2
7	401	1369±494	3.4±1.2
7	432	1369±494	3.2±1.1
7	467	1369±494	2.9±1.1
7.1	825	1365±492	1.7±0.6

7.1	777	1365±492	1.8±0.6
7.5	276	1342±485	4.9±1.8
8	247	1311±475	5.3±1.9
8	293	1311±475	4.5±1.6
8	300	1311±475	4.4±1.6
8	382	1311±475	3.4±1.2
8	390	1311±475	3.4±1.2
8	415	1311±475	3.2±1.1
8	475	1311±475	2.8±1
8	524	1311±475	2.5±0.9
8	586	1311±475	2.2±0.8
8	605	1311±475	2.2±0.8
8	623	1311±475	2.1±0.8
8	793	1311±475	1.7±0.6
8	457	1311±475	2.9±1
8.1	679	1308±474	1.9±0.7
8.2	623	1298±471	2.1±0.8
8.4	804	1290±468	1.6±0.6
9	347	1255±457	3.6±1.3
9	267	1254±457	4.7±1.7
9	344	1254±457	3.6±1.3
9	378	1254±457	3.3±1.2
9	391	1254±457	3.2±1.2
9.2	518	1245±454	2.4±0.9
9.3	782	1236±451	1.6±0.6
9.5	375	1226±448	3.3±1.2
9.8	398	1208±442	3±1.1
9.9	262	1203±440	4.6±1.7
9.9	372	1202±440	3.2±1.2
9.9	406	1201±440	3±1.1
10	136	1196±438	8.8±3.2
10	236	1196±438	5.1±1.9
10	348	1196±438	3.4±1.3
10	358	1196±438	3.3±1.2
10	500	1196±438	2.4±0.9
10	401	1194±437	3±1.1
10.1	382	1190±436	3.1±1.1
11	300	1141±420	3.8±1.4
11	237	1138±420	4.8±1.8
11	296	1138±420	3.8±1.4
11	310	1138±420	3.7±1.4
11	328	1138±420	3.5±1.3
11	353	1138±420	3.2±1.2
11	370	1138±420	3.1±1.1
11	398	1138±420	2.9±1.1
11	406	1138±420	2.8±1
11	417	1138±420	2.7±1
11	435	1138±420	2.6±1
11	537	1138±420	2.1±0.8
11.2	550	1126±415	2±0.8
11.4	293	1116±412	3.8±1.4

11.5	339	1111±411	3.3±1.2
12	193	1081±401	5.6±2.1
12	301	1081±401	3.6±1.3
12	314	1081±401	3.4±1.3
12	406	1081±401	2.7±1
12	640	1081±401	1.7±0.6
12	406	1080±401	2.7±1
13	223	1023±383	4.6±1.7
13	257	1023±383	4±1.5
13	430	1023±383	2.4±0.9
13	441	1023±383	2.3±0.9
13.3	441	1006±377	2.3±0.9

Table S3. Accuracy of estimates of R_0. For the 25 children in Table 1, we compare the R_0 values estimated with the approximate estimation based simply on the CD4 T cell counts at treatment initiation. Estimates of CD4 counts in healthy individuals of the same age are obtained using the linear expression in Fig. S4. The corresponding approximate estimates are obtained by dividing the latter CD4 counts with the baseline CD4 counts in the infected children. Note that the differences between the two estimates exist, if at all, in the second decimal place, implying less than 1% deviation between the two.

**Patient ID**	**Age (y)**	**Baseline CD4 count (cells/µl)**	**Estimated CD4 counts in age-matched healthy individuals (cells/µl)**	**Estimate of R_0 from Table 1**	**Approximate estimate of R_0**
2	9.9	372	1201.77	3.23	3.23
3	9	347	1253.7	3.62	3.61
4	7.5	276	1340.25	4.86	4.86
7	2.5	2706	1628.75	0.60	0.60
8	6.9	244	1374.87	5.66	5.63
9	10	401	1196	2.98	2.98
10	7	415	1369.1	3.30	3.30
11	9.9	406	1201.77	2.96	2.96
13	9.5	375	1224.85	3.27	3.27
15	11	300	1138.3	3.80	3.79
16	9.9	262	1201.77	4.60	4.59
18	6.6	1366	1392.18	1.02	1.02
19	9.8	398	1207.54	3.04	3.03
21	4.9	514	1490.27	2.90	2.90
23	8.2	623	1299.86	2.09	2.09
24	3.1	1737	1594.13	0.92	0.92
25	10.1	382	1190.23	3.12	3.12
27	8.4	804	1288.32	1.60	1.60
30	9.2	518	1242.16	2.40	2.40
31	9.3	782	1236.39	1.58	1.58
32	7.1	777	1363.33	1.75	1.75
33	8.1	679	1305.63	1.92	1.92
34	9	391	1253.7	3.21	3.21
35	6.9	615	1374.87	2.24	2.24
36	12	193	1080.6	5.60	5.60

Table S4. Standard errors estimated for the fit parameters. Standard errors associated with the best-fit parameters for the children in Table 1 are reported here.

PID*	A	δ (/d)	δ_M (/d)
2	1.51×10^{-10}	8.61×10^{-14}	8.83×10^{-15}
3	2.79×10^{3}	1.19×10^{-1}	3.32×10^{-3}
4	1.25×10^{2}	1.90×10^{-2}	1.36×10^{-3}
8	3.93×10^{2}	6.01×10^{-2}	2.50×10^{-3}
9**	1.58×10^{3}	1.48×10^{-1}	4.19×10^{-3}
10	3.50×10^{2}	1.14×10^{-2}	1.28×10^{-3}
11	8.30×10^{1}	4.32×10^{-2}	6.70×10^{-3}
15	4.27×10^{-11}	1.80×10^{-15}	6.84×10^{-16}
18	1.68×10^{3}	3.16×10^{-1}	4.15×10^{-3}
21	2.11×10^{-11}	1.92×10^{-15}	4.93×10^{-16}
23	6.98×10^{1}	2.20×10^{-2}	3.34×10^{-3}
24	4.94×10^{2}	1.25×10^{-1}	3.97×10^{-3}
25	1.80×10^{-11}	1.98×10^{-15}	5.06×10^{-16}
27	1.20×10^{-11}	7.52×10^{-15}	4.29×10^{-16}
31	5.59×10^{-11}	7.25×10^{-15}	2.64×10^{-15}
33	1.12×10^{-10}	5.09×10^{-14}	1.70×10^{-14}
34	3.55×10^{2}	3.86×10^{-2}	3.54×10^{-3}
35**	6.96×10^{3}	4.54×10^{-2}	5.86×10^{-2}

*PIDs 7, 13, 16,19, 30, 32 and 36 were excluded due to paucity of measurements (only 3 viral load measurements were available). For PIDs 2, 15, 21, 25, 27, 31, and 33, there were typically 4 viral load measurements each, leading to near perfect fits, and hence low uncertainties in best-fit estimates.

**Estimation of standard errors for PID 9 and 35 was not robust. Hence, we fixed either δ or δ_M to the mean value across children and estimated the uncertainty in the other.

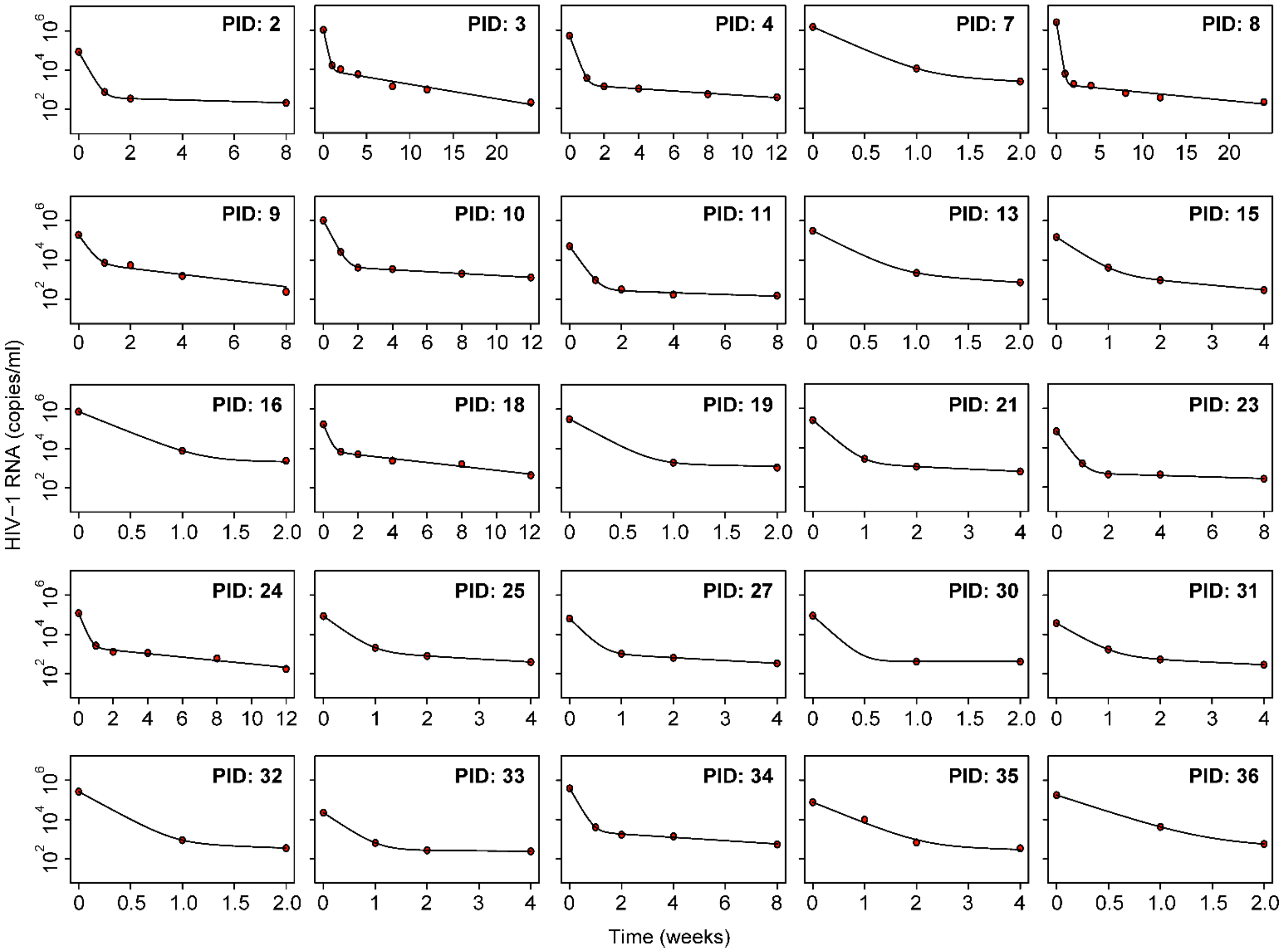

Figure S1. Model fits to data. Best-fits of model predictions (black lines) to viral load decay data (red circles) for the 25 children considered.

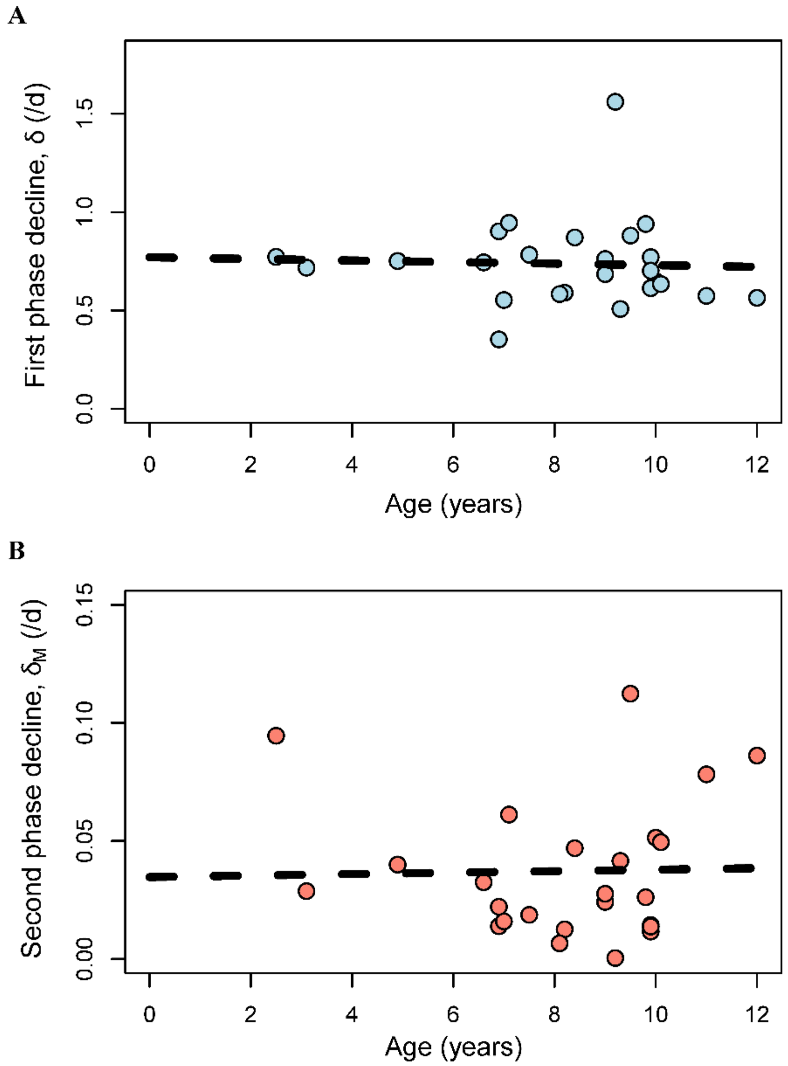

Figure S2: Dependence of viral load decay slopes on age. (A) Correlation of the first phase decline rate, δ, with age. Pearson ρ=-0.04 (P=0.85). (B) Correlation of the second phase decline rate, δ_M, with age. Pearson ρ=0.024 (P=0.91). The estimates of δ and δ_M were obtained from the fits of our mathematical model of viral dynamics to patient data (n=25, Fig. S1). Dashed lines are the respective best-fit lines.

A

B

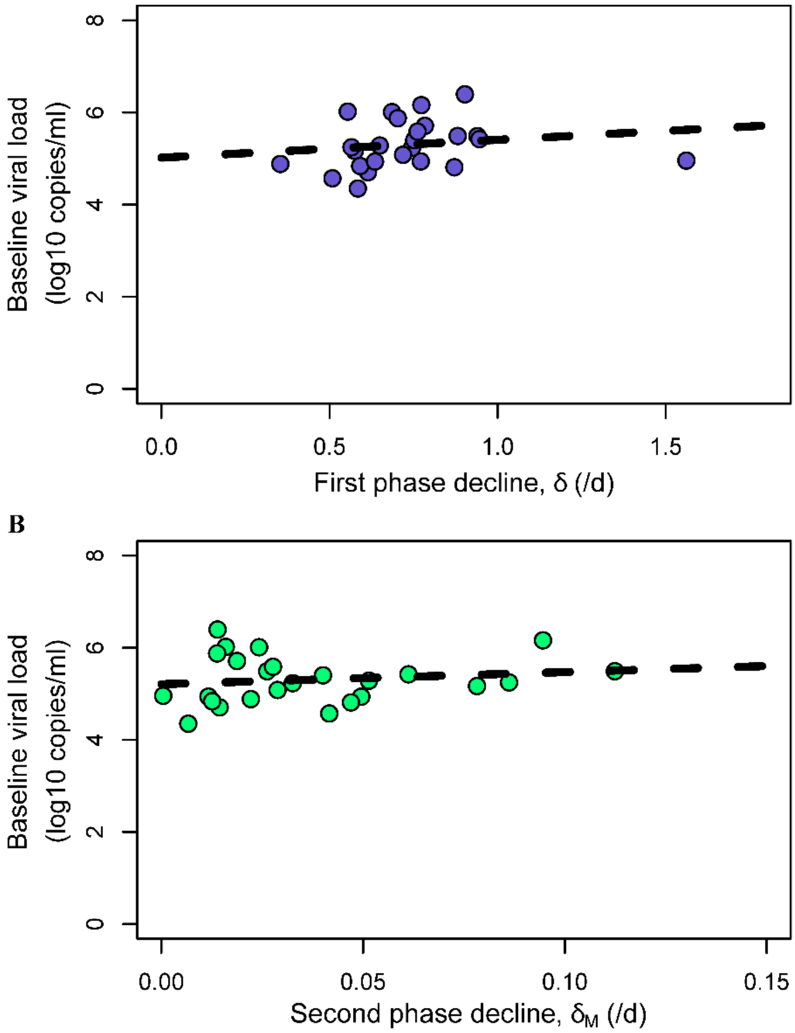

Figure S3: Correlation of the baseline viral load with decay rates (A) Correlation of the measured baseline viral load with the first phase decline, δ. Pearson ρ=0.167 (P=0.43). (B) Correlation of the measured baseline viral load with second phase decline, δ_M. Pearson ρ=-0.148 (P=0.48). The estimates of δ and δ_M were obtained from the fits of our mathematical model of viral dynamics to patient data (n=25, Fig. S1). Dashed lines are the respective best-fit lines.

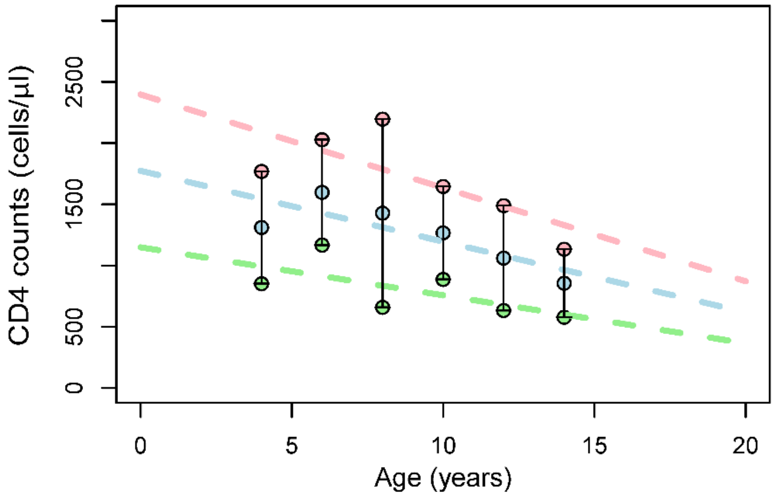

Figure S4: Dependence of CD4 T cell counts on age in healthy children. Mean CD4 T cell counts (blue symbols; Ref. [20]) decline linearly (blue line) with age in healthy children in India. The best-fit yielded $CD4(a) = 1773 - 57.7a$ (see text). Similar fits to the counts 1 SD above (pink symbols) and below (green symbols) the mean yielded $CD4(a) = 2396 - 76.2a$ (pink line) and $CD4(a) = 1150 - 39.2a$ (green line).

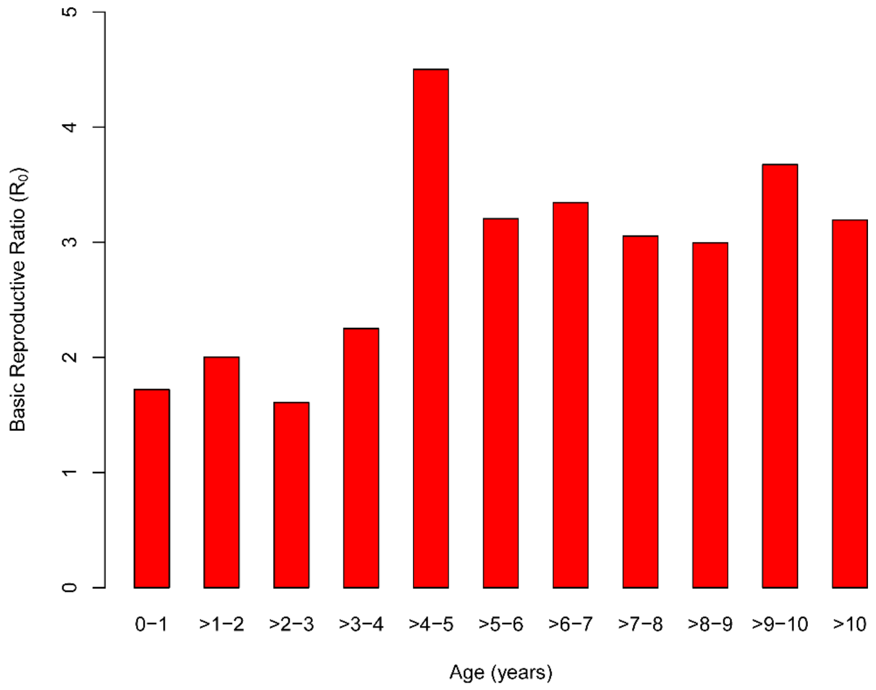

Figure S5: Age stratified *R_0*. Estimates of R_0 from all children with baseline CD4 T cell counts above 100 cells/µl (n=157, Fig. 3B) stratified in 1 y age bands.

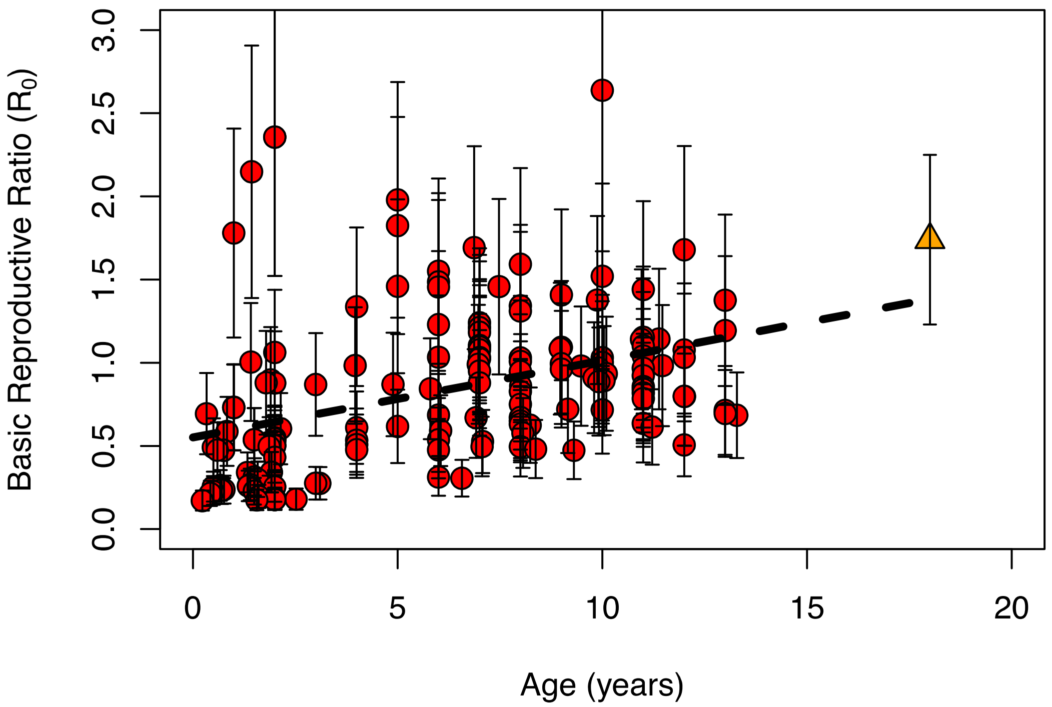

Figure S6: Estimates of R_0 accounting for reduction in thymic output. We obtained estimates of R_0 from all children as in Fig. 3B but now with 30% reduced CD4 T cell counts in uninfected individuals. The other details are the same as in Fig. 3B. These are likely to be underestimates of R_0 given the partial restoration of CD4 counts following therapy, the extent of which remains unclear. The trends of increasing R_0 with age remain (Pearson ρ=0.38 (P=9.1×10^{-7})) and the estimates converge to the value associated with adults.
